# Rate of spontaneous onset of labour before planned repeat caesarean section at term

**DOI:** 10.1186/1471-2393-14-125

**Published:** 2014-04-03

**Authors:** Christine L Roberts, Michael C Nicholl, Charles S Algert, Jane B Ford, Jonathan M Morris, Jian Sheng Chen

**Affiliations:** 1 Clinical and Population Perinatal Health Research, Kolling Institute, University of Sydney, Sydney, New South Wales, Australia; 2 Department of Obstetrics and Gynaecology, University of Sydney, Sydney, New South Wales, Australia; 3 Department of Obstetrics and Gynaecology, Royal North Shore Hospital, St Leonards, New South Wales, Australia

**Keywords:** Cohort study, Elective repeat caesarean section, Labour, Record linkage

## Abstract

**Background:**

Guidelines recommend that, in the absence of compelling medical indications (low risk) elective caesarean section should occur after 38 completed weeks gestation. However, implementation of these guidelines will mean some women go into labour before the planned date resulting in an intrapartum caesarean section. The aim of this study was to determine the rate at which low-risk women planned for repeat caesarean section go into spontaneous labour before 39 weeks.

**Methods:**

We conducted a population-based cohort study of women who were planned to have an elective repeat caesarean section (ERCS) at 39-41 weeks gestation in New South Wales Australia, 2007-2010. Labour, delivery and health outcome information was obtained from linked birth and hospital records for the entire population. Women with no pre-existing medical or pregnancy complications were categorized as ‘low risk’. The rate of spontaneous labour before 39 weeks was determined and variation in the rate for subgroups of women was examined using univariate and multivariate analysis.

**Results:**

Of 32,934 women who had ERCS as the reported indication for caesarean section, 17,314 (52.6%) were categorised as ‘low-risk’. Of these women, 1,473 (8.5% or 1 in 12) had spontaneous labour or prelabour rupture of the membranes before 39 weeks resulting in an intrapartum caesarean section. However the risk of labour <39 weeks varied depending on previous delivery history: 25% (1 in 4) for those with *spontaneous* preterm labour in a prior pregnancy; 15% (1 in 7) for women with a prior *planned* preterm birth (by labour induction or prelabour caesarean) and 6% (1 in 17) among those who had only previously had a planned caesarean section at term. Smoking in pregnancy was also associated with spontaneous labour. Women with spontaneous labour prior to a planned CS in the index pregnancy were at increased risk of out-of-hours delivery, and maternal and neonatal morbidity.

**Conclusions:**

These findings allow clinicians to more accurately determine the likelihood that a planned caesarean section may become an intrapartum caesarean section, and to advise their patients accordingly.

## Background

For planned caesarean section among low risk women, birth at 39 weeks gestation or later has demonstrated benefits for the mother and baby [1-4]. Accordingly, guidelines recommend that in the absence of compelling medical indications, elective caesarean section should occur after 38 completed weeks gestation [5-8]. However, implementation of these guidelines will mean some women go into labour before the planned date resulting in an intrapartum caesarean section. Intrapartum caesarean section is associated with increased risk of maternal and neonatal morbidities and has resource implications, especially if the procedure occurs outside usual operating room times [9,10]. Furthermore, obstetricians report that women always ask about the likelihood of going into labour before their planned caesarean date. This is particularly pertinent to women who live in rural and remote areas who may need to move closer to maternity unit around the expected date of birth.

Knowing the likelihood of spontaneous labour prior to planned caesarean section at term can aid clinicians, women and maternity units in delivery and service planning but little evidence is available. A rate of 12% spontaneous labour at 37-38 weeks for women before a planned “nonemergent elective caesarean section” at 39-41 weeks can be derived from an American single-hospital study conducted in 1980 [11]. More recent literature suggests that 10% of women booked for caesarean section at 39 weeks will labour prior to the scheduled date, [6,8,12] but this rate has been extrapolated indirectly from gestational age distributions at term not determined directly. Therefore, the primary aim of this study was to determine the overall rate of spontaneous labour before 39 weeks among a contemporary population of low-risk women planned for an elective repeat caesarean section at term. A secondary aim was to identify subgroups at greater or lesser risk of spontaneous labour.

## Methods

### Study population and data sources

The study population was women giving birth in New South Wales (NSW), Australia, who had caesarean section at term (37-41 weeks) where the indication was elective repeat caesarean section (ERCS) and resulted in a singleton live birth. With a resident population of nearly 7 million people, NSW is the most populous state of Australia. NSW has approximately 95,000 births per annum (one-third of all Australian births), of which fewer than 0.5% are home births.

In this study, we used information from two linked population health datasets for the period from 1 January 2007 to 31 December 2010: the New South Wales (NSW) Perinatal Data Collection (PDC) and the NSW Admitted Patient Data Collection (APDC). The PDC records all births in NSW of at least 20 weeks gestation or at least 400 grams birth weight. Information recorded by the midwife/medical practitioner in the PDC includes maternal demographic characteristics and information on maternal health, pregnancy, labour, delivery, and infant outcomes. The APDC is a census of all NSW inpatient hospital discharges (public and private) and includes demographic and episode-related data; diagnoses and procedures are coded for each admission from the medical records according to the 10th revision of the International Statistical Classification of Diseases and Related Health Problems, Australian Modification (ICD-10-AM) and the affiliated Australian Classification of Health Interventions [13]. Record linkage of the PDC and APDC (including mothers’ and babies’ hospital admissions) and longitudinal linkage of pregnancies (to establish obstetric history) was undertaken by the NSW Centre for Health Record Linkage [14]. As Australia does not have a unique registration number for citizens, the separate datasets were linked using probabilistic linkage methods and a best practice approach in preserving privacy [14,15]. This involves a process of blocking and matching combinations of selected variables such as name, date of birth, address and hospital and assigning a probability weight to the match [16]. The validity of probabilistic record linkage is extremely high; [17] for this study, the CHeReL reported the quality of the record linkage as 3 per 1,000 false positive links and <5 per 1,000 missed links. The researchers were provided anonymised data. Ethics approval for the study was obtained from the NSW Population and Health Services Research Ethics Committee.

**Figure 1:**
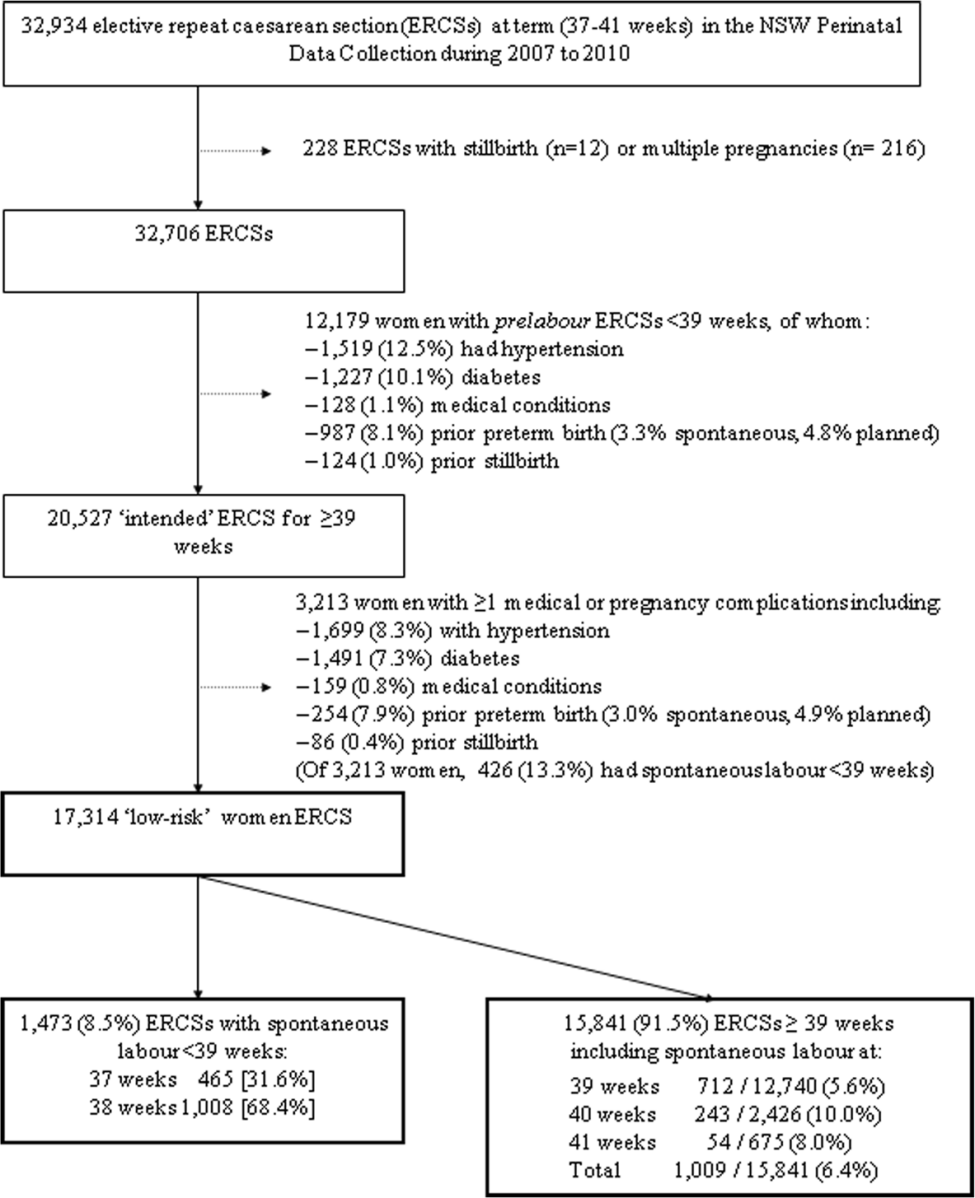
Study population flow diagram.

In the PDC, the main indication for caesarean section is reported by a single-option check-box as: failure to progress, fetal distress, clinical indication (such as hypertension or other medical conditions), ERCS or non-clinical indication (4%; includes maternal choice but excludes repeat CS) [18]. We selected women where the main indication at the time of birth was ERCS, and where the woman was delivered by either prelabour or intrapartum caesarean. As the main interest was in spontaneous onset of labour (including prelabour rupture of the membranes [PROM]) in uncomplicated pregnancies planned for 39 weeks gestation, women with prelabour ERCS at 37-38 weeks (n = 12,179) were excluded from the study (Figure 1). The remainder were considered ‘intended’ ERCS at ≥ 39 weeks. Women having an ERCS may have other co-morbidities that influence the decision for and timing of ERCS such as hypertension (gestational, preeclampsia or chronic), gestational or pre-gestational diabetes, other medical conditions (including cardiac, renal, thyroid or autoimmune disease) or a previous stillbirth. Women with these conditions are not the target of elective caesarean guidelines [5-8] and were excluded from the main analysis (Figure 1); the remainder were considered ‘low-risk ERCS’. Gestational age is reported in the PDC in completed weeks of gestation and is based on the best clinical estimate including early (<20 weeks’ gestation) ultrasound scan (>97%) and last menstrual period.

The primary outcome was spontaneous onset of labour (referred to as spontaneous labour), and secondary outcomes were ‘out of hours’ birth (between 7 pm and 7 am, during weekends or on NSW public holidays), use of general anaesthesia for caesarean section, postpartum haemorrhage, and severe (potentially life-threatening) maternal and neonatal morbidity/mortality using validated composite indicators [19,20]. The Neonatal Adverse Outcome Indicator (NAOI) includes mortality and a comprehensive list of procedures and diagnoses indicating severe neonatal morbidity, such as mechanical ventilation, respiratory distress syndrome, parenteral nutrition, sepsis and hypoxic ischemic encephalopathy [19]. Maternal morbidity and mortality were measured using a similar validated composite indicator relating to serious adverse maternal health outcomes such as transfusion, pulmonary embolism, hysterectomy, and mechanical ventilation [20]. Composite indicators overcome the problem of under-ascertainment of individual adverse events and reduce the need to rely on single ICD codes that have limited clinical detail, lack clear definitions or are poorly validated [20].

Factors of interest in examining subgroups at risk of spontaneous labour before 39 weeks included maternal and pregnancy characteristics: maternal age (categorized as <20, 20-34 and ≥35 years), country of birth (Australia and New Zealand, Asia and other countries), socio-economic status based on residential postcode and grouped into quintiles according to the Australian Bureau of Statistics ‘Index of Relative Socio-economic Disadvantage’, [21] smoking during pregnancy, parity (including number of previous caesarean sections and vaginal births), previous spontaneous labour, previous preterm birth, fetal presentation at birth, gestational and sex adjusted birth weight (<10^th^ percentile, 10^th^-90^th^ percentile and >90^th^ percentile); [22] and maternity care characteristics: type of hospital (private, tertiary obstetric with or without neonatal intensive care, other urban and rural public), type of care within hospital (private or public), and antenatal care (commenced <20 or ≥20 weeks gestational weeks). Previous delivery history for each woman was categorized, with respect to spontaneous labour and/or preterm birth, by a prespecified descending hierarchy of clinical relevance: i) any preterm birth following spontaneous onset of labour, ii) any planned preterm birth (following a decision to deliver by caesarean section or induction of labour), iii) any term birth following spontaneous onset of labour, or iv) only term planned birth(s).

### Data analysis

Descriptive statistics were used to summarize the distributions of maternal and pregnancy characteristics among women with low-risk ERCS who had spontaneous labour before 39 gestational weeks and those who birthed at 39-41 weeks. Regression models were employed to identify characteristics associated with either increased or decreased risk of spontaneous labour before 39 weeks. In the multivariable analyses, a backwards elimination approach was used to progressively remove the least significant terms until all terms remaining were statistically significant (*P* <0.05, two-sided). Crude and adjusted relative risks (RR) were estimated using log-binomial regression. Other than prior delivery history, few records had missing data (<4%). Prior delivery history was not available for women who had previously delivered outside the state or study period. Due to a larger number of women missing prior delivery history (14%), they were retained in the regression analysis as a group but denoted as delivery history ‘unavailable’. Other records with missing data were excluded from the multivariable analysis. To explore the role of gestation at a prior birth, a subgroup analysis was conducted using only the women whose prior delivery history information was available. To assess the pattern of association of prior delivery history, we plotted the probability of spontaneous labour before 39 weeks at ERCS against the earliest gestational age in prior births for each of 3 prior delivery scenarios (ie any prior spontaneous labour, only prior labour induction of labour and only prior prelabour caesarean sections). The secondary outcomes were used to assess any extra burden on the health care system if low-risk women ‘intended’ for ERCS ≥39 weeks had spontaneous labour before 39 weeks. Differences in secondary outcomes between women with spontaneous labour at 37-38 weeks and those with ERCS at 39-41 weeks were compared using the chi-square test and unadjusted risks are used to quantify the population health services burden.

## Results

From 2007 to 2010, 32,934 women had ERCS at term as the reported indication for CS. Excluding 12,179 women delivered by prelabour caesarean at 37-38 weeks as well as 228 multiple gestations and stillbirths, 20,527 (62.3%) were included as “intended” ERCS for ≥39 weeks (Figure 1). Of these, 17,314 (52.6%) were categorised as ‘low-risk’ (no pre-existing medical or pregnancy complications and not delivered by prelabour caesarean section at 37-38 weeks).

The 17,314 low-risk women ‘intended’ for ERCS ≥39 weeks delivered at 89 hospitals with a median of 31 such deliveries per hospital per annum (inter-quartile range 11 to 71). Of these, 1,473 (8.5%) had spontaneous labour or PROM before 39 weeks (2.7% at 37 weeks and 5.8% at 38 weeks), and a further 4.5% had spontaneous labour at 39 weeks. The risk of spontaneous onset of labour before 39 weeks was higher (13.3%, Figure 1) for women with co-morbidities (RR of spontaneous labour 1.56, 95% CI 1.41-1.72 compared to low-risk women).

Table 1 presents the distribution of maternal characteristics and pregnancy factors among low-risk women with spontaneous labour before 39 weeks, compared to those who had their caesarean as planned. Women who were born in Asia, smokers or had 2 or more previous CS were more likely to go into spontaneous labour before 39 weeks. The rate of spontaneous labour before 39 weeks by risk factors highlights the importance of prior delivery history (Table 2). For example, low-risk women who had spontaneous preterm labour in a previous pregnancy had a much higher rate of spontaneous labour before 39 weeks compared to women who had only ever had planned delivery at term (25.4% vs 5.9%). Factors significant in the univariate analysis (Asian-born, smoked during pregnancy, had a prior preterm delivery [either following spontaneous labour or planned], spontaneous labour at term in a previous pregnancy or more than one previous caesarean section) remained significant in a multivariable regression model (Table 2). The risk for women whose prior delivery history (n = 2,488, 14.4%) was unavailable was intermediate (adjusted RR 1.39) consistent with this group including women from all categories. The findings were unchanged when the multivariable analysis was restricted to the women whose delivery history information was available.

**Table 1:** **Maternal**, **pregnancy and maternity care characteristics of low risk women for elective repeat caesarean section** (**ERCS**)

	**Spontaneous labour <39 weeks N = 1**,**473 N (Col.%)**^**a**^	**ERCS** ≥**39 weeks N = 15**,**841 N (Col.%)**^**a**^	**P value**
**Maternal and Pregnancy Characteristics**			
**Age** (years)			0.87
<20	10 (0.7)	96 (0.6)	
20 - <35	922 (62.6)	10,005 (63.2)	
> = 35	541 (36.7)	5,739 (36.2)	
**Country of birth**			<0.001
Australian	1,013 (68.9)	11,650 (73.7)	
Asian	258 (17.6)	1,980 (12.5)	
Other	199 (13.5)	2,177 (13.8)	
**Socio**-**economic status**			
Most disadvantaged	293 (20.1)	2,851 (18.2)	0.24
Disadvantaged	254 (17.5)	2,763 (17.7)	
Average	263 (18.1)	2,734 (17.5)	
Advantaged	259 (17.8)	3,082 (19.7)	
Most advantaged	386 (26.5)	4,210 (26.9)	
**Smoking in pregnancy**	176 (12.0)	1,385 (8.7)	<0.001
**No. of previous vaginal births**			0.16
0	1,256 (87.1)	13,716 (88.8)	
1	133 (9.2)	1,261 (8.2)	
≥2	53 (3.7)	478 (3.1)	
**No. of previous CS**			0.001
1	1,013 (70.2)	11,513 (74.5)	
2	358 (24.8)	3,315 (21.5)	
≥3	73 (5.1)	630 (4.1)	
**Prior delivery history**^**b**^			<0.001
Spontaneous preterm	102 ( 6.9)	300 (1.9)	
Planned preterm	65 ( 4.4)	382 (2.4)	
Spontaneous term	570 (38.7)	4,685 (29.6)	
Planned term	512 (34.8)	8,210 (51.8)	
Unavailable	224 (15.2)	2,264 (14.3)	
**Vertex Presentation**	1,440 (97.8)	15,471 (97.7)	0.82
**Size at birth**			0.03
<10^th^ percentile	81 (5.5)	822 (5.2)	
10^th^ - 90^th^ percentile	1,194 (81.1)	12,480 (78.8)	
>90^th^ percentile	197 (13.4)	2,532 (16.0)	
**Maternity Care Characteristics**			
**Care type** (private)	700 (47.5)	7,624 (48.1)	0.66
**Antenatal care** (<20w)	1,390 (94.4)	14,963 (94.5)	0.88
**Birth hospital type**			
Private	533 (36.2)	6,064 (38.3)	0.19
Tertiary	483 (32.8)	5,260 (33.2)	
Other (urban)	225 (15.3)	2,184 (13.8)	
Other (rural)	232 (15.8)	2,333 (14.7)	

^a^Percentages may not add to 100 because of missing data.

^b^Incorporates spontaneous onset of labour or planned delivery (by labour induction or prelabour caesarean section) for the lowest gestation of all prior births.

**Table 2:** **Among low risk women for elective repeat caesarean section** (**ERCS**), **the factors associated with spontaneous labour at 37**-**38 weeks**

	**Total low risk women N** **=** **17**,**314**	**Spontaneous labour <39 weeks N** **=** **1**,**473**		**Crude RR (95% CI)**	**Adjusted RR**^**b **^**(95% CI)**
		N	Rate (Row%)^a^		
**Age** (years)					
<20	106	10	9.4	1.12 (0.62, 2.02)	
20 - <35	10,927	922	8.4	1.00	
> = 35	6,280	541	8.6	1.02 (0.92, 1.13)	
**Country of birth**					
Australian	12,663	1,013	8.0	1.00	1.00
Asian	2,238	258	11.5	1.44 (1.27, 1.64)	1.53 (1.33, 1.75)
Other	2,376	199	8.4	1.05 (0.91, 1.21)	1.09 (0.94, 1.26)
**Socio**-**economic status**					
Most disadvantaged	3,144	293	9.3	1.00	
Disadvantaged	3,017	254	8.4	0.90 (0.77, 1.06)	
Average	2,997	263	8.8	0.94 (0.80, 1.10)	
Advantaged	3,341	259	7.8	0.83 (0.71, 0.98)	
Most advantaged	4,596	386	8.4	0.90 (0.78, 1.04)	
**Smoking in pregnancy**	1,561	176	11.3	1.37 (1.18, 1.59)	1.33 (1.14, 1.56)
**No. of previous vaginal births**					
0	14,972	1,256	8.4	1.00	1.00
1	1,394	133	9.5	1.14 (0.96, 1.35)	0.94 (0.80-1.12)
≥2	531	53	10.0	1.19 (0.92, 1.54)	0.89 (0.68-1.15)
**No. of previous CS**					
1	12,562	1,013	8.1	1.00	1.00
2	3,673	358	9.8	1.21 (1.07, 1.35)	1.16 (1.04, 1.30)
≥3	703	73	10.4	1.28 (1.03, 1.61)	1.16 (0.92, 1.45)
**Prior delivery history**^**c**^					
Spontaneous preterm	402	102	25.4	4.31 (3.57, 5.20)	4.12 (3.38, 5.03)
Planned preterm	447	65	14.5	2.49 (1.96, 3.16)	2.47 (1.94, 3.14)
Spontaneous term	5,255	570	10.8	1.85 (1.65, 2.08)	1.85 (1.65, 2.07)
Planned term	8,722	512	5.9	1.00	1.00
Unavailable	2,488	224	9.0	1.54 (1.32, 1.79)	1.39 (1.19, 1.63)
**Vertex Presentation**	16,911	1,440	8.5	1.04 (0.75, 1.45)	
**Size at birth**					
<10^th^ percentile	903	81	9.0	1.03 (0.83, 1.27)	
10^th^ - 90^th^ percentile	13,674	1,194	8.7	1.00	
>90^th^ percentile	2,729	197	7.2	0.83 (0.72, 0.96)	
**Care type** (private)	8,324	700	8.4	0.98 (0.89, 1.08)	
**Antenatal care** (<20w)	16,353	1,390	8.5	0.98 (0.80, 1.22)	
**Birth hospital type**					
Private	6,597	533	8.1	0.96 (0.85, 1.08)	
Tertiary	5,743	483	8.4	1.00	
Other (urban)	2,409	225	9.3	1.11 (0.96, 1.29)	
Other (rural)	2,565	232	9.0	1.08 (0.93, 1.25)	

^a^Unadjusted rate of spontaneous labour <39 weeks for each characteristic.

^b^Adjusted for other variables in the column.

^c^Incorporates spontaneous onset of labour or planned delivery (by labour induction or prelabour caesarean section) for the lowest gestation of all prior births.

**Figure 2:**
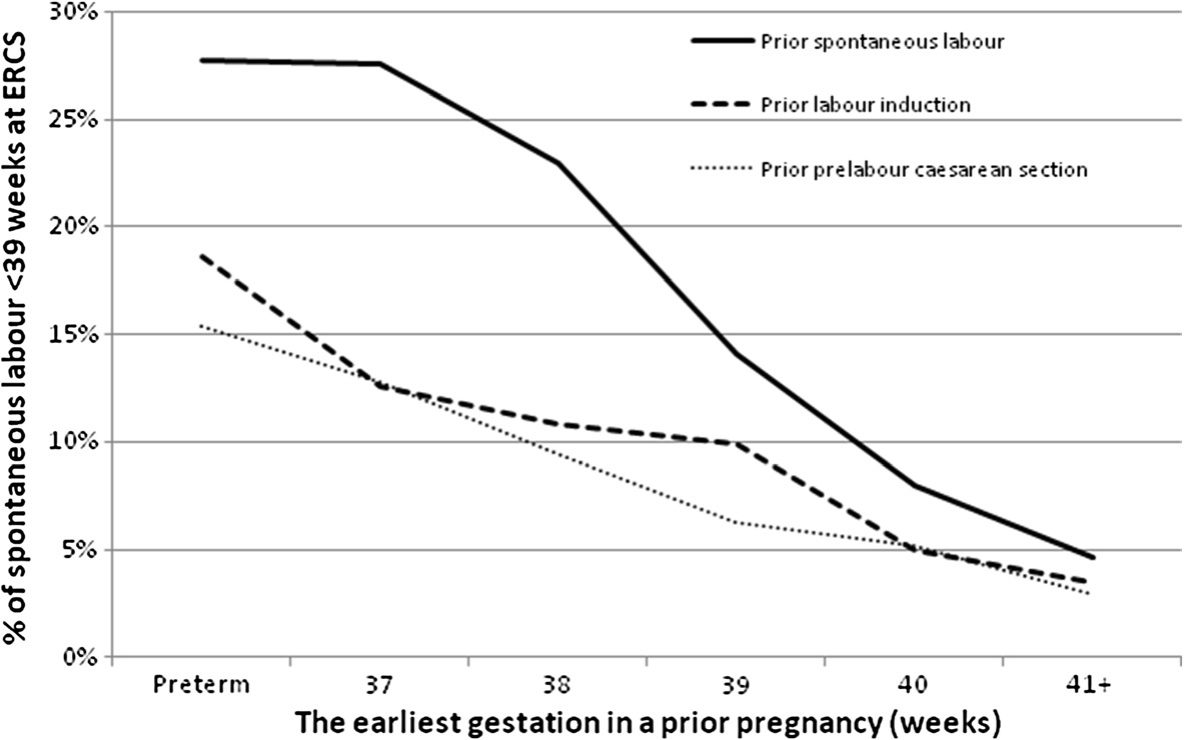
**Association between gestational age and labour onset in a prior birth**, **and the probability of spontaneous labour before 39 weeks at ERCS.**

Among the 14,829 (86%) low-risk women ‘intended’ for ERCS ≥39 weeks who had an available delivery history, gestational age at a prior birth showed an inverse association with the probability of spontaneous labour before 39 weeks. This pattern was strongest for prior births with spontaneous onset of labour, but was also observed following previous labour induction and prelabour caesarean (Figure 2).

Compared to low-risk women with ERCS ≥39 weeks, those with spontaneous labour <39 weeks were, unsurprisingly, more likely to have an out of hours caesarean section (60.8% vs 11.2%; RR = 5.43 95% CI 5.11-5.77, P <0.001). They also had an increased risk of severe maternal morbidity (2.0% vs 0.8%; RR = 2.56, 95% CI 1.71-3.82, P <0.001) and neonatal morbidity (2.2% vs 1.0%; RR = 2.23 95% CI 1.53-3.26; P <0.001); but non-significant increases in the use of general anaesthesia (8.0% <39 weeks vs 6.8% ≥39 weeks; RR 1.18 95% CI 0.98-1.42; P =0.08) and postpartum haemorrhage (3.8% <39 weeks vs 3.0% ≥39 weeks; RR = 1.26, 95% CI 0.96-1.65; P =0.10).

## Discussion

Using contemporary Australian data, we estimate an overall 8.5% risk (1 in 12) of intrapartum caesarean before 39 weeks for low-risk women having an elective repeat caesarean section. This rate is lower than previously reported. Importantly, the overall figure masks the variable impact of previous births. If low-risk women have no history of preterm birth and/or spontaneous labour in a previous pregnancy, the risk is only 6% (1 in 17). Spontaneous labour and/or rupture of membranes prior to 39 weeks is likely to occur in at least 1 in 7 women with prior preterm birth and 1 in 9 women with prior spontaneous labour at term. The likelihood of spontaneous onset is also increased if women had previously experienced a preterm birth, were born in Asia, were smokers or had two or more caesarean births previously. For women with spontaneous preterm labour in any previous pregnancy (2.3% of low risk women for ERCS) the risk of labour before a subsequent scheduled caesarean is quadrupled and occurred in 1 in 4 of these women.

Knowing the likelihood of spontaneous labour prior to planned caesarean section at term can be useful in planning the place and time of birth, especially in countries like Australia where women in rural and remote communities are recommended to move close to their maternity hospital when birth is imminent. For example, a 25% risk of intrapartum caesarean section (because of spontaneous preterm labour in a previous pregnancy) might affect the advice given, and even result in a decision to plan delivery before the recommended 39 weeks.

This population-based study utilizes recent data, reflecting current obstetric practice. We focused on low-risk women intended for delivery at ≥39 weeks, as this is the group of most interest for policies targeting reductions in non-medically indicated early term delivery [5-8]. Almost half of the women who had ERCS recorded as the main indication for caesarean section had co-morbidities that may have influenced decision-making (Figure 1). Co-morbidity and prior preterm birth rates were highest for women who had an ERCS at 37-38 weeks. So although these women appear to have received care that was contrary to the guideline recommendation, it may might reflect a decision to act early because of a perceived increase in the risk of spontaneous labour before 39 weeks. A US study found that when up to three indications for CS could be recorded (from 20 options, including ‘other’), approximately 30% of women who had a repeat caesarean delivery at term had potential medical indications for caesarean delivery [4]. This likely reflects complex (rather than single option) decision-making, and underscores the importance of identifying pregnancies that would not be subject to gestational age recommendations for the timing of low-risk caesarean section. Routine data collection on indications for caesarean section may be better served by allowing the selection of multiple indications for CS [23]. Furthermore, a recent observed trend towards later gestation among prelabour caesarean sections at term, subsequent to introduction of a policy requiring that elective caesarean section should not occur prior to 39 completed weeks gestation, may mask an even greater change in the timing of elective caesareans among low-risk women [24].

To our knowledge the risk factors for spontaneous onset of labour at term but before 39 weeks have not been previously assessed, so comparison with other studies is not possible and replication of the findings of this study is desirable. Spontaneous labour in a prior pregnancy as a predictor of subsequent spontaneous labour is consistent with clinical expectation. In contrast to the lack of information at term, there is extensive literature on risk factors for spontaneous *preterm* birth. As the focus of this study was low risk women, many women with risk factors for shorter gestations were excluded (e.g. women with multiple pregnancies, prior stillbirth, medical conditions and pregnancy complications). However, consistent with the preterm literature we found smoking, prior preterm birth (especially with spontaneous onset), extremes of maternal age and low SES to be risk factors for spontaneous labour <39 weeks [25-27].

The increased risk of unplanned labour for Asian born women was unexpected as several studies have reported Asian women to have lower rates of preterm birth and the biological plausibility of the gestational age and ethnicity association is unclear [28-30]. Although this does not preclude differences in the onset of labour at term in the subgroup of low risk women with a previous caesarean section, the finding needs to be interpreted with caution as Asian ethnicity is identified only by self-reported country of birth. Similarly there is no obvious clinical explanation for the slightly increased rate of spontaneous labour prior to 39 weeks for women with two or more prior caesarean sections. This may be a chance finding from a large dataset or alternately may reflect a lower threshold at which clinicians diagnose ‘labour” when a woman presents with uterine activity with a history of two or more caesarean sections.

Compared to women who reached 39 weeks gestation, the rates of severe maternal and neonatal morbidity were higher among the women with spontaneous labour <39 weeks, consistent with studies showing a stepwise decline in adverse outcomes as gestation advances [2,31]. Some of the increased neonatal morbidity could be due to intrapartum caesarean as opposed to prelabour caesarean, but some would be due to the consequences of not reaching full fetal maturity. Additionally, the risk of adverse outcomes may be confounded by the reason for spontaneous onset of labour even in the apparently uncomplicated pregnancies. The findings from this study are consistent with previous studies showing that at or close to term, the benefit of each increased week of gestation is greater than the respiratory benefit of fetal exposure to labour [2,32]. Each additional week of gestation does present some risk of intrauterine death, however for births where the infant is ≥10^th^ percentile for size the risk of intrauterine death at 37 and 38 weeks is less than 2 per 10,000 ongoing pregnancies [33].

A natural consequence of spontaneous labour <39 weeks was the increase in ‘out of hours’ caesarean section and a tendency towards increased rates of general anaesthesia. Out of hours caesarean section has resource implications for operating theatre availability as well as staffing needs including obstetricians, midwives, anaesthetists and other operating theatre staff.^5^ This may be especially acute in low-volume district and private hospitals which often require theatre staff to be called in after hours. The observed general anaesthesia rate is lower than reported in 2004 for this population [34], consistent with application of guidelines recommending regional block for most caesarean births [6]. That the difference in general anaesthesia use between intrapartum and prelabour caesareans did not meet statistical significance is consistent with widespread availability of regional anaesthesia for maternity care [18].

Although we observed a slightly higher postpartum haemorrhage rate (3.8%) for women with spontaneous onset of labour <39 weeks, it was not significantly different to those with caesarean ≥39 weeks (3.0%). Previous studies found women with a previous or planned caesarean to be at significantly increased risk of postpartum haemorrhage and transfusion when the caesarean was intrapartum rather than prelabour [35,36]. Importantly, the latter studies were not restricted to low-risk women and the magnitude of the increased risk in these studies (≥30%) was similar to our non-significant results (RR of 1.26).

The strengths of the study are the use of large, linked population health datasets that include one-third of all births in Australia. The data utilised in this study have been shown to be accurate and reliable when validated against medical records [37-39]. Furthermore, with the exception of detailed information on prior delivery history, missing values were infrequent. The results are generalisable to all Australian women and possibly other high income countries. Although the impact of variable local practices is uncertain, ERCS is undoubtedly of increasing importance. In many countries ERCS currently consitutes around one-third of all CS but the contribution is increasing as the primary CS rate increases [40-42]. As in many studies utilising large datasets for entire populations, it is important to consider whether statistically significant differences are clinically meaningful. Finally, information was not available on other factors that have been demonstrated to be associated with shorter gestation such as short inter-pregnancy interval, body mass index and poor nutritional status [27].

## Conclusions

In summary, the risk of spontaneous labour before a planned CS is strongly influenced by prior delivery history. Weighing up the risk that a planned caesarean may become an intrapartum caesarean is part of the decision-making about when to schedule delivery, and underscores the importance of obtaining a complete pregnancy history.

## Competing interests

The authors declare that they have no competing interests.

## Authors’ contributions

CLR, MCN and JMM conceived the study and JSC coordinated the project. All authors participated in the study design, planning of analysis and interpretation of the results. JSC undertook the data preparation, statistical analyses and provided statistical expertise. CLR, CSA and JSC drafted the manuscript, JFB provided epidemiological expertise, and MCN and JMM provided clinical expertise. All authors critically reviewed drafts of the manuscript, and read and approved the final manuscript.

## Pre-publication history

The pre-publication history for this paper can be accessed here:

http://www.biomedcentral.com/1471-2393/14/125/prepub
